# Factors associated with the effectiveness and reach of NHS stop smoking services for pregnant women in England

**DOI:** 10.1186/s12913-017-2502-y

**Published:** 2017-08-08

**Authors:** L. R. Vaz, T. Coleman, S. J. Fahy, S. Cooper, L. Bauld, L. Szatkowski, J. Leonardi-Bee

**Affiliations:** 1 UK Centre for Tobacco and Alcohol Studies, Division of Primary Care, University of Nottingham Medical School, Queen’s Medical Centre, Nottingham, NG7 2RD UK; 2 Institute for Social Marketing, UK Centre for Tobacco and Alcohol Studies, University of Stirling, Stirling, FK9 4LA UK; 3 UK Centre for Tobacco and Alcohol Studies, Division of Epidemiology and Public Health, University of Nottingham, Nottingham City Hospital, Clinical Sciences Building 2, Hucknall Road, Nottingham, NG5 1PB UK

**Keywords:** Smoking, Pregnancy, Stop smoking services

## Abstract

**Background:**

The UK National Health Service provides Stop Smoking Services for pregnant women (SSSP) but there is a lack of evidence concerning how these are best organised. This study investigates influences on services’ effectiveness and also on their propensity to engage pregnant smokers with support in stopping smoking.

**Methods:**

Survey data collected from 121/141 (86%) of SSSP were augmented with data from Hospital Episode Statistics and the 2011 UK National Census. ‘Reach’ or propensity to engage smokers with support was defined as the percentage of pregnant smokers setting a quit date with SSSP support, and ‘Effectiveness’ as the percentage of women who set a quit date who also reported abstinence at four weeks later. A bivariate (i.e. two outcome variable) response Markov Chain Monte Carlo model was used to identify service-level factors associated with the Reach and Effectiveness of SSSP.

**Results:**

Beta coefficients represent a percentage change in Reach and Effectiveness by the covariate. Providing the majority of one-to-one contacts in a clinic rather than at home increased both Reach (%) (β: 6.97, 95% CI: 3.34, 10.60) and Effectiveness (%) (β: 7.37, 95% CI: 3.03, 11.70). Reach of SSSP was also increased when the population served was more deprived (β for increase in Reach with a one unit increase in IMD score: 0.55, 95% CI: 0.25, 0.85), had a lower proportion of people with dependent children (β: -2.52, 95% CI: -3.82, −1.22), and a lower proportion of people in managerial or professional occupations (β: -0.31, 95% CI: -0.59, −0.03). The Effectiveness of SSSP was decreased in those areas that had a greater percentage of people >16 years with no educational qualifications (β: -0.51, 95% CI: -0.95, −0.07).

**Conclusions:**

To engage pregnant smokers and to encourage them to quit, it may be more efficient for SSSP support to be focussed around clinics, rather than women’s homes. Reach of SSSP is inversely associated with disadvantage and efforts should be made to contact these women as they are less likely to achieve abstinence in the short and longer term.

## Background

Smoking during pregnancy is a public health problem of international concern. In high-income countries large numbers of women smoke when pregnant; prevalence is reported as 30–35% in Spain [1] and in 2010, 26% of UK women smoked in pregnancy, with 12% smoking continuously throughout gestation. [2] Similar estimates have been reported in Canada and Japan (10%), [3, 4] with a slightly higher prevalence in the USA (14%). [5] There are lower rates in low and middle income countries but these are increasing such that the World Health Organisation (WHO) describes a developing epidemic of smoking in pregnancy in these jurisdictions. [6] Conception is a life event which strongly motivates smokers towards cessation, with around 50% of smokers trying to stop during pregnancy. [7] Sustained smoking abstinence which begins in pregnancy not only benefits smokers; it very likely also improves their children’s health. Smoking during pregnancy is associated with increased risk of low birthweight and preterm birth. [8] However, while children of non-smokers are less likely to become smokers; [9] the impact of maternal smoking on future child smoking appears to be exerted after childbirth, rather than as a result of ‘in utero’ effects [10].

Counselling [11] and self-help [12] interventions are effective for helping pregnant smokers to stop and the former also improve adverse birth outcomes. [11] However, few countries systematically offer smoking cessation support to pregnant women, despite this being consistent with Article 14 of the Framework Convention on Tobacco Control, [13] the international public health treaty which has been adopted by WHO. The UK is one country which offers no-cost smoking cessation support to all pregnant women; such support is provided by local stop smoking services (SSS) which are available nationally. Individuals are able to access one-to-one support, behavioural support, pharmacotherapy and incentives depending upon availability at the local SSS. SSS for all smokers were first introduced in 1999, and from 2000, tailored SSS which serve pregnant smokers were funded. Although stop smoking services for pregnancy (SSSP) are now mature components of the UK National Health Service (NHS), there is only limited evidence to guide their provision. In comparison, UK SSS for non-pregnant smokers have been evaluated such that, short [14] and longer term outcomes [15] are known and outcome variations have been investigated. [16] In the non-pregnant population, group support helped a greater number of smokers to quit at four weeks, and SSS in more deprived areas reached more people, but SSS that operated in these more disadvantaged areas had lower cessation rates than those in more affluent areas [16].

However, as pregnancy is a unique life event, with potentially differing motivations for quitting and physiological strength of addiction, one cannot assume that the evidence for services aimed at non-pregnant smokers applies to SSSP. Pregnant smokers are often more disadvantaged, and those who are disadvantaged are often less likely to receive necessary care. [17] Consequently, we used a detailed survey to describe key variations in English SSSP. [18] Here survey information is combined with census and routinely-available data on smoking in pregnancy and on SSSP performance to determine the SSSP features which are associated with attracting pregnant smokers into treatment and with the outcomes of support.

## Methods

### Data sources

Data for this study were derived from a survey of SSSP, [18] routinely reported stop smoking service performance data [19] and Hospital Episode Statistics (HES). [20] In addition, data from the 2011 UK National Census, aggregated at the SSSP level, was used to determine, within each SSSP area, the proportions of people who were not of ‘white’ or ‘mixed’ ethnicity; who were ≥16 years old with no qualifications; who had children; were working in managerial or professional occupations; and were <18 years of age. [21] The average index of multiple deprivation (IMD) score for the location the SSSP was situated in was taken from statistics compiled by the UK Government. [22] The IMD is a composite measure that combines indicators of deprivation across seven different domains: income, employment, health and disability, education, crime, barriers to housing and services, and living environment. A higher score is associated with increased levels of deprivation.

The survey of SSSP was designed and implemented using Qualtrics software (Qualtrics Labs Inc., Provo, UT) and asked about the period between April 2010 and March 2011. The online survey was originally piloted in 14 services and pilot responses were analysed along with the responses from other SSSP. An online survey link was sent to managers of all 141 SSSP services in England via email and non-responders were contacted via email after three to four months. If after this, no response was received, contact was made via telephone and a hard copy, electronic copy or a copy via telephone was completed. Services that completed this (*N* = 121, 86%) tended to support more pregnant women and provide more effective cessation support than non-respondent services. [18] The original survey found that there were no systematic differences between responding and non-responding SSSP to the survey with respect to area level IMD or self-reported quit rates [18]. However, respondent services were more likely to have greater number of women set quit dates and to have quit by four weeks. [18] Carbon monoxide validation was also more complete in respondent SSSP and there was borderline evidence (*p* = 0.05) that respondent SSSP were more likely to have smoking status at time of delivery. [18]

A full list of independent variables included in the analyses and the sources from which these were derived is detailed in Table 1; this also includes the rationale for each variable’s inclusion and, where available, references to justify why these were considered to have the potential to influence outcomes. Where references are not used alongside justifications in Table 1, variables were included on the basis of the authors’ opinion.

**Table 1:** Variables considered for inclusion in the model

Independent variable	Description, source & justification	Dependent variable tested against
The mean index of multiple deprivation (IMD)	Continuous variable. SSSP level IMD was used. IMD score ranged from 9 to 43, with higher score indicating greater deprivation. Taken from UK Data Service Census Support. [22] Measures of deprivation were inversely associated with pregnant smokers’ cessation in a recent large RCT. [34] SSS for non-pregnant smokers have successfully attracted smokers from lower socio economic groups. [31]	Reach, [31] Effectiveness [34]
Survey data		
Whether or not mandatory midwifery training in local hospitals included smoking cessation	Binary variable. SSSP in which midwifes received smoking cessation training versus those who did not. It was hypothesised that training could influence propensity for midwives to refer women to SSSP and initial midwife contacts may be more effective at promoting smoking cessation.	Reach, Effectiveness
Whether or not identification of smokers used CO monitoring and an opt-out referral pathway	Binary variable. SSSP in which the opt-out referral pathway was used versus those that did not. This kind of referral pathway has been shown to increase SSSP referrals (and hence Reach) [35] and has been associated with better service outcomes in an observational study. [36]	Reach, [35] Effectiveness [36]
Whether or not the Commissioning for Quality and Innovation (CQUIN) framework [37] was in place for referrals to the SSSP	Binary variable. SSSP in which either the CQUIN was in place for recording smoking status, referrals, providing brief advice or any other, versus not being so. We hypothesised that having such a local policy in place which encouraged identification of smokers could result in greater Reach.	Reach
Whether the SSSP used social networking sites to engage women	Binary variable. SSSP in which specialist cessation service offered was advertised using any social networks. We hypothesised that services which pro-actively attempted to engage with women in this way may have greater Reach.	Reach
Whether SSSP staff initially contacted referred pregnant smokers by phone	Binary variable. SSSP that initially contacted women via phone as opposed to letter, SMS, email, face-to-face contact, home visit or if pregnant women contacted the service. We hypothesised that the manner of initial contact could have consequences for Reach.	Reach
Whether the majority of one-to-one support offered by the SSSP was at home or in the clinic	Binary variable. SSSP that offered more behavioural support at home as opposed that offered in the clinic. We hypothesised that offering support at home might influence Reach; there is evidence that for non-pregnant smokers, location of support provision can influence the Effectiveness of support delivered. [38]	Reach, Effectiveness [38]
Whether the SSSP centre offered couple or family support	Binary variable. SSSP that provided behavioural support in the form of couple or family support versus those that did not. We hypothesised that offering partner support might increase the likelihood of women attending SSSP (Reach); partner support is a key variable influencing pregnant women’s success in cessation attempts. [39]	Reach, Effectiveness [39]
Whether there were any financial incentives offered by the SSSP	Binary variable. SSSP that provided an incentive scheme for pregnant women versus those that did not. We hypothesised that the availability of incentives might affect women’s propensity to engage with services; a recent trial has shown these to be effective in the SSSP context. [40]	Reach, Effectiveness [40]
Whether self-referral patients accounted for one of the top three referral methods to the SSSP	Binary variable. SSSP in which self-referral was at least the third most popular referral method, versus those in which it was not. We hypothesised that flexibility in accepting referrals might affect Reach.	Reach
Whether the SSSP offered ‘dual therapy’ nicotine replacement therapy (NRT) (i.e. longer and shorter acting preparations used together)	Binary variable. SSSP that offered dual therapy NRT with or without behavioural support versus SSSP that did not. Although there is no evidence that standard dose NRT works we hypothesised that higher doses from use of ‘dual therapy’ might be effective.	Effectiveness
Census data		
In the absence of a strong evidence base, all of the following variables were considered to have the potential to affect either Effectiveness or Reach		
The proportion of women in the area covered by the SSSP not of white or mixed ethnicity	Continuous variable. National census data aggregated at the SSSP level. [21] We hypothesised women who did not identify as white or mixed ethnicity might be less likely to smoke and may be less likely to seek help to quit smoking.	Effectiveness, Reach
The proportion of people aged ≥16 years in the area covered by the SSSP with no qualifications	Continuous variable. National census data aggregated at the SSSP level. [21] We hypothesised that the level of education is inversely associated with the odds of cessation, and may also be associated with awareness of harms and thus, making a quit attempt.	Effectiveness, Reach
The proportion of people in the area covered by the SSSP with dependent children	Continuous variable. National census data aggregated at the SSSP level. [21] We hypothesised that having dependent children may decrease the odds of being able to attend clinic visits.	Effectiveness, Reach
The proportion of people in the area covered by the SSSP in managerial or professional occupations	Continuous variable. National census data aggregated at the SSSP level. [21] We hypothesised women who are in managerial or professional occupations may have higher levels of education, and be less likely to smoke. They may view themselves as less likely to need help and not attend SSSP.	Effectiveness, Reach
The proportion of people in the area covered by the SSSP aged <18 years	Continuous variable. National census data aggregated at the SSSP level. [21] We hypothesised being of younger age may affect women’s likelihood of quitting smoking.	Effectiveness, Reach

### Derivation of dependant variables, ‘reach’ and ‘effectiveness’

#### Reach

A measure called ‘Reach’ was developed to provide an estimate of SSSP success in engaging pregnant smokers to access cessation support. This was defined as the percentage of all pregnant smokers residing within an area served by a SSSP who, with SSSP support, set a date for quitting smoking: data on whether or not clients set quit dates with SSSP support were routinely collected by SSSPs. The numerator for this measure was taken from SSSP statistics routinely reported quarterly to the UK Department of Health (DH); all stop smoking services (SSS) tell the DH the numbers of smokers who have set ‘quit dates’ with SSS support. [23] Data for the number of pregnant smokers in areas served by SSSP are not routinely available and so were derived using a synthetic estimation procedure. [24] This involved building a multilevel logistic regression model using data from the 2010 Infant Feeding Survey (IFS) to estimate the probability of a pregnant woman smoking at any point during her pregnancy according to her age, IMD quintile, and the ethnicity profile of the area in which she lived. [24] To estimate the number of smokers in any one SSSP area, with Bayesian 95% Credible Intervals, these probabilities were applied to numbers of women with these socio-demographic characteristics giving birth in that area, derived from 2010/11 HES. Further details about this synthetic estimation procedure, and discussion of the validity of the estimates, have been published elsewhere [24].

#### Effectiveness

Our Effectiveness measure was designed to reflect SSSP success in helping smokers achieve abstinence in any one quit attempt; this was defined as the percentage of those pregnant smokers who set a quit date with SSSP support who reported abstinence four weeks later. The numerator and the denominator were taken from NHS SSS statistics for April 2010 to March 2011, which are routinely reported to DH [23].

### Analysis strategy

#### Linear regressions and model building

Univariable linear regressions were carried out between the outcomes of ‘Reach’ and ‘Effectiveness’ and the predictor variables (Table 1). Variables which showed significant (*p* ≤ 0.1) associations in the univariable models were initially included in a bivariate response model in which both ‘Reach’ and ‘Effectiveness’ were modelled jointly. In building a parsimonious model with as few predictors as possible, variables found to be non-significant in the bivariate response model were then removed; this minimised the variance of the model. Following this, all remaining predictor variables were tested individually, beginning with those most strongly associated with either Reach or Effectiveness, to investigate whether they became significant if added to the bivariate response model. A bivariate response model was used as the error terms in the equations for Reach and Effectiveness were hypothesised to be correlated, i.e. Reach and Effectiveness are associated, and by using this kind of model, we hoped to produce more precise estimates.

#### Missing values and imputation

Missing data for predictor variables listed in Table 1 were imputed by Multiple Imputation using Chained Equations (MICE). An imputation model was constructed based on selected predictor variables and the two outcome variables, Reach and Effectiveness. The imputation model was based on 10 imputed datasets [25] and an indicator variable was included for clustering at the SHA level.

#### IGLS model for deriving starting parameter estimates for bivariate model

An Iterative Generalised Least Squares (IGLS) model using maximum likelihood estimates was used as an initial estimation method to provide non-informative priors for a model using Markov Chain Monte Carlo methods (MCMC). The analysis strategy, previously outlined in the ‘linear regressions and model building’ section above, was used to create the IGLS model. At the time of the survey, SSSPs were commissioned by local Primary Care Trusts (PCTs) which were clustered within larger Strategic Health Authorities (SHA); the public health team within each SHA advised PCTs on the delivery of SSSP and thus one could expect some similarities between SSSPs delivered by PCTs in the same SHA. To account for this clustering, a random effects model was used with clustering at the level of the SHA.

#### MCMC estimating parameter estimates for bivariate model

A MCMC method using Gibbs Sampling method was employed to provide model estimates. [26] MCMC methods are used to efficiently sample from a complicated distribution, such as a bivariate model for Reach and Effectiveness of SSSP. A burn-in (i.e. period which allows the Markov chains to converge) of 500 iterations was used to achieve an equilibrium distribution, after which these were discarded. A further 10,000 iterations were then performed. [26] The trajectories of these simulations were checked for each variable to confirm that they converged to a random scatter around a stable mean. Stable simulations beyond the burn-in were used to characterise the posterior distribution i.e. estimates from the 10,000 iterations that were stable were used to calculate the estimates for variables in the model. The effective sample size (ESS) was maximised using hierarchical centring which improved the efficiency of the simulation and reduced the effect of autocorrelation (i.e. an iterations’ result is dependent upon its place in the sequence or related to other iterations). ESS represents the independent draws the sample represents for each parameter compared to the 10,000 iterations performed. The larger the effective sample size, the better the posterior distribution is sampled using MCMC, and less likely iterations are to be autocorrelated. Further information on the iterative MCMC procedure is published elsewhere [26].

The fit of the variables in the MCMC estimation was summarised using the Bayesian Deviance Information Criterion (DIC). Bayesian DIC decreases with increasing complexity of the model, so as the model increases in size, variables added are less likely to be considered to make a significant contribution to the model fit. A new DIC was calculated after adding a variable to the model; if the DIC decreased by more than two points, this indicated that the model was a significantly better fit with that variable included. [27] The MCMC estimation produced point estimates with 95% Credible Intervals.

Additionally, the Variance Partition Coefficient (VPC) was also employed to measure the amount of clustering by SHA. This measured the proportion of the variation in Reach and Effectiveness that was explained by SSSPs being located in different SHAs, and helped to indicate whether changes in factors at the SHA level might influence the two outcomes.

#### Sensitivity analysis

To assess whether any bias had been introduced into the model, a sensitivity analysis was conducted comparing the model presented, which used multiple imputation for missing data in predictor variables, with a comparator model that used list-wise deletion for handling these missing data. A second sensitivity analysis was also carried out comparing the point estimates of the synthetic estimates of the SSSP outcome variable, ‘Reach’, with the associated 95% Credible Intervals. This was done to assess whether differences in the parameter estimates of Reach resulted in changes to the model produced.

All analyses were carried out using Stata 13.1 (College Station, TX), MLwiN Version 2.30 (Centre for Multilevel Modelling, University of Bristol) [28] and the ‘runmlwin’ Stata command [29].

#### Ethical approval and consent

This secondary analysis of data collected was conducted in accordance within the ethical tenants of the declaration of Helsinki (as revised in 1983). Ethics approval was not required for data from the SSSP survey as it was obtained as part of a service evaluation. Written consent was received from the practice managers who provided data for the SSSP survey. Routinely collected smoking data did not require ethical approval as this was derived from the publicly available UK national census. However, as the Health & Social Care Information Centre (HSCIC) makes HES and UK census data available for research with the proviso of proper use, all data were used in line with HSCIC policy of protecting the confidentiality and privacy of individuals.

#### Availability of data and materials

Data used for this study were derived from a survey of SSSP, [18] routinely reported stop smoking service performance data [19] and Hospital Episode Statistics (HES) [20]. Stop smoking service and HES data are available from their respective cited sources. Data from the survey of SSSP is not available.

## Results

121/141 (86%) of SSSP provided useable survey responses. The majority of SSSP provided services for a single PCT with only 10 SSSP commissioned by more than one PCT; consequently, we did not account for clustering of SSSP within PCTs. The mean Reach of SSSP (percentage of pregnant smokers in area served by SSSP setting a quit date) was 17.2% (SD: 12.5%, *n* = 121) and the mean Effectiveness (percentage of pregnant smokers who set a quit date and reported cessation at 4 weeks) was 45.2% (SD: 11.4%, *n* = 121). Using data that were complete we were able to impute values for those predictor variables that had missing data. Outcomes could not be calculated for two SSSP (Isle of Man, Kensington & Chelsea) because data on the number of quit dates was not available. Therefore, after multiple imputation, subsequent analysis was carried out in 139 SSSP.

The results of the univariable linear regressions demonstrated that having the majority of support delivered at a clinic was associated with increased Reach and Effectiveness of SSSP (Tables 2 and 3). Both Reach and Effectiveness of a SSSP were increased in areas where there were more people over the age of 16 years with no qualifications and in areas with a greater percentage of people with dependent children. Thus, all variables that were associated with Effectiveness were also associated with Reach.

**Table 2:** Linear regression associations with “Reach”

	N (%)/ mean		95% CI		
Variable	[SD]^a^	β^b^	Lower	Upper	*p*-value
*Mean IMD score for SSSP* ^c^	23.27 [8.09]	0.38	0.17	0.59	0.004
*Did employees mandatory midwifery training include smoking cessation?*					
No	48 (41.4)	ref	-	-	-
Yes	68 (58.6)	2.59	−1.10	6.28	0.247
*Did identification of smokers use both CO monitoring and the opt-out referral pathway?*					
No	92 (79.3)	ref	-	-	-
Yes	24 (20.7)	−0.51	−5.18	4.16	0.855
*Was the Commissioning for Quality and Innovation (CQUIN) framework in place for referrals to the SSSP?*					
No	85 (78.0)	ref	-	-	-
Yes	24 (22.0)	−2.14	−6.88	2.60	0.453
*Did the SSSP used social networking site to engage women?*					
No	86 (81.1)	ref	-	-	-
Yes	20 (18.9)	2.94	−1.88	7.76	0.313
*Did the SSSP initially contacted pregnant women by phone?*					
No	14 (12.1)	ref	-	-	-
Yes	102 (87.9)	−1.82	−7.80	4.17	0.615
*Was the majority of one-to-one support offered by the SSSP in the clinic?*					
No	57 (53.8)	ref	-	-	-
Yes	49 (46.2)	8.97	5.55	12.38	<0.001
*Did the SSSP centre offer couple or family support?*					
No	60 (56.1)	ref	-	-	-
Yes	47 (43.9)	−0.22	−4.27	3.84	0.929
*Were financial incentives offered by the SSSP?*					
No	70 (64.8)	ref	-	-	-
Yes	38 (35.2)	4.38	0.46	8.30	0.067
*Did the SSSP centre currently have a specialist advisor?*					
No	27 (22.7)	ref	-	-	-
Yes	92 (77.3)	10.77	6.86	14.68	<0.001
*Did self-referral patients account for one of the top three referral methods to the SSSP?*					
No	48 (41.7)	ref	-	-	-
Yes	67 (58.3)	2.55	−1.21	6.31	0.263
*Women who did not identify as white or mixed ethnicity women in the area covered by SSSP* ^c^ *(%)*	13.6 [14.9]	−0.27	−0.38	−0.15	<0.001
*People aged ≥ 16 years with no qualifications* ^c^ *(%)*	22.8 [4.9]	1.11	0.79	1.44	<0.001
*People with dependent children* ^c^ *(%)*	64.1 [1.9]	−1.69	−2.60	−0.77	0.003
*People in managerial or professional occupations* ^c^ *(%)*	36.1 [8.4]	−0.61	−0.80	−0.41	<0.001
*People aged < 18 years* ^c^ *(%)*	18.4 [3.1]	0.65	0.07	1.23	0.064

^a^Counts and means prior to multiple imputation (*N* = 121)

^b^Regression coefficients based on 139 SSSPs, using results from imputed datasets

^c^Regression results for mean centred variables

**Table 3:** Linear regression associations with “Effectiveness”

	N (%)/ mean		95% CI		
Variable	(SD)^a^	β	Lower	Upper	*p*-value
*Mean IMD score for SSSP* ^b^	23.27 [8.09]	−0.09	−0.29	0.11	0.445
*Did employees mandatory midwifery training include smoking cessation?*					
No	48 (41.4)	ref	-	-	-
Yes	68 (58.6)	1.25	−2.38	4.88	0.567
*Did identification of smokers use both CO monitoring and the opt-out referral pathway?*					
No	92 (79.3)	ref	-	-	-
Yes	24 (20.7)	−0.10	−4.34	4.14	0.969
*Was the majority of one-to-one support offered by the SSSP in the clinic?*					
No	57 (53.8)	ref	-	-	-
Yes	49 (46.2)	6.62	3.12	10.12	0.002
*Did the SSSP centre offer couple or family support?*					
No	60 (56.1)	ref	-	-	-
Yes	47 (43.9)	−3.07	−6.65	0.50	0.157
*Were financial incentives offered by the SSSP?*					
No	70 (64.8)	ref	-	-	-
Yes	38 (35.2)	0.42	−3.59	4.44	0.860
*Did the SSSP centre currently have a specialist advisor?*					
No	27 (22.7)	ref	-	-	-
Yes	92 (77.3)	0.49	−3.33	4.32	0.831
*Did the SSSP offer dual therapy NRT?*					
No	13 (12.0)	ref	-	-	-
Yes	95 (88.0)	−1.78	−6.75	3.19	0.553
*Women who did not identify as white or mixed ethnicity women in the area covered by SSSP* ^b^ *(%)*	13.6 [14.9]	0.08	−0.03	0.19	0.227
*People aged ≥ 16 years with no qualifications* ^b^ *(%)*	22.8 [4.9]	−0.51	−0.84	−0.19	0.009
*People with dependent children* ^b^ *(%)*	64.1 [1.9]	0.88	0.03	1.73	0.088
*People in managerial or professional occupations* ^b^ *(%)*	36.1 [8.4]	0.13	−0.06	0.32	0.270
*People aged < 18 years* ^b^ *(%)*	18.4 [3.1]	−0.48	−1.01	0.04	0.132

^a^Counts and means prior to multiple imputation

^b^Regression results for mean centred variables

Reach was also increased in SSSPs that served a more deprived (higher IMD score) population, offered financial incentives, had a specialist advisor who had received some training in smoking cessation, and in areas where a greater percentage of people were aged <18 years. Reach of SSSP was inversely associated with the percentage of people of who did not identify as white or mixed ethnicity and the percentage in managerial or professional occupations.

The parameter estimates for the predictor variables modelled jointly for Reach and Effectiveness are presented in Table 4. The equation for Reach of SSSP explained 38.0% of the variation and the equation for Effectiveness explained 15.0% of the variation. For each unit increase in IMD, representing greater deprivation within an area, there was a 0.55% increase in Reach of SSSP indicating that pregnant women residing in areas that were considered more deprived were more likely to be reached by SSSP. Providing one-to-one support at a clinic rather than in women’s homes was associated with a 6.97% increase in the proportion of pregnant smokers setting a quit date (i.e. Reach) and associated with a 7.37% increase in the proportion of women reporting smoking cessation at four weeks post-quit date (i.e. Effectiveness). For every 1% increase in the percentage of people with dependent children or of people in managerial or professional occupations residing in areas served by SSSP, there was a 2.52% and 0.31% decrease in SSSP Reach, respectively. Every 1% increase in the percentage of people aged 16 or older that had no qualifications residing in an SSSP catchment area was associated with a 0.51% decrease in SSSP Effectiveness.

**Table 4:** MCMC estimates

		95% CI		Difference in DIC	
Variable	β	Lower	Upper		ESS
Reach					
*Mean IMD score* ^a,b^	0.55	0.25	0.85	−11.59	7002
*Majority of one-to-one support at clinic*	6.97	3.34	10.60	−13.10	8798
*Percentage of people with dependent children* ^a^	−2.52	−3.82	−1.22	−12.19	5259
*Percentage of people in managerial or professional occupations* ^a^	−0.31	−0.59	−0.03	−3.10	4896
Effectiveness					
*Majority of one-to-one support at clinic*	7.37	3.03	11.70	−13.99	9035
*Percentage of people* ≥ *16 years with no qualifications* ^a^	−0.51	−0.95	−0.07	−2.63	5281

^a^mean centred variables

^b^higher score = more deprived

The VPC indicated that total proportion of the variance that was explained by differences at the SHA level in the model for Reach and Effectiveness was 15.2% and 12.0% respectively; thus, the majority of variance, and hence the majority of variation in SSSP performance, was explained at the SSSP level rather than at SHA level. The effective sample size was >1000 for each of the variables included in the model indicating an efficient simulation.

### Sensitivity analysis

The sensitivity analysis which investigated differences in findings for the binary response model based on whether the missing data was imputed or deleted in a list-wise manner found no significant differences in results obtained using these two strategies (results available upon request). Additionally, findings from a sensitivity analysis which compared the point estimates used for Reach with those from the associated 95% Credible Intervals found that the estimates for the predictor variables included in the multivariable linear regression models did not change substantially (results available upon request).

## Discussion

This study has identified that the setting used by the English smoking cessation services for providing cessation support in pregnancy (SSSPs) may affect important service outcomes; when SSSPs primarily helped women from clinic settings rather than through home visits, they not only reached more smokers, but their support was more effective too. Additionally we have shown how local population characteristics might affect SSSP outcomes. Being located in areas with greater material deprivation, where fewer people have dependent children and fewer people work in managerial occupations was associated with SSSPs reaching and helping a higher proportion of local pregnant smokers; English SSSP services effectively target disadvantaged groups. However, being in areas with more non-qualified 16 year olds was inversely associated with SSSPs effectiveness; once using the SSSPs, pregnant women from more disadvantaged areas are less likely to quit.

This is the first study to investigate how the design of nationally-provided services for smoking cessation in pregnancy and the characteristics of women using these might affect service outcomes. Information sources were robust; UK census data and small area statistics are of very high quality; routinely-collected outcome data were available for almost all SSSPs and the survey which provided data on service characteristics had a high response rate. Together these factors suggest that findings are likely to be relevant to SSSPs within the English NHS and, potentially generalisable, to similar services serving high-income countries where smoking in pregnancy is concentred amongst materially deprived women. Findings have face validity rather than being counter-intuitive. One would expect area and service organisational characteristics to influence SSSP ability to ‘Reach’ smokers more than efficacy of SSSP interventions delivered. Also, as expected, these explained much more of the variance in SSSPs’ ability to reach pregnant smokers than they did in SSSP Effectiveness.

A limitation is that our analyses did not include individual-level data on service users; instead, small area census data have been used as a proxy. Systematic differences between women accessing SSSPs and the female population of the areas in which they lived could affect study findings. Although there is no reason to suppose that systematic differences existed, findings are still probably best used to inform us on key organisational issues that affect service performance, rather than on what influence clients’ characteristics might have. As analyses were observational, we cannot claim a causal relationship between factors identified as potentially-important and the two outcomes. As much variance in these was not explained by our models, it is possible that important influences on service outcomes were not captured by data sources used in analyses. Consequently, there may be other, as yet undescribed factors, which also contribute to SSSP performance. Finally, findings may only generalise to countries with a similar economic profile to England’s; in jurisdictions in which the relationship between deprivation and smoking in pregnancy is weaker, there may be different influences on women’s smoking behaviour in pregnancy and hence the ability of health service interventions to influence this.

The finding that the provision of clinic-based support was associated with SSSPs helping more pregnant smokers to stop is important. In 2011, presumably to encourage pregnant smokers to access SSSP, 73% of English services offered resource-intensive home support visits. [18] This rationale appears incorrect since this study demonstrates clinic-based support was associated with both increased rates of reach (setting a quit date) and effectiveness (abstinence rates), which could be used to argue for using clinics rather than women’s homes as the primary location from which to offer support.

However, it is also possible that the apparently greater effectiveness of clinic based support could be due to women with higher motivation to quit making the effort to attend clinic appointments. Amongst non-pregnant smokers, greater motivation to stop is associated with better attendance at SSS appointments [30] and motivation is a key determinant of success in smoking cessation attempts. Health services have little influence on the underlying characteristics of the populations they serve, but services do need to be configured to serve populations appropriately and analysis outcomes are reassuring on this point. As for SSS that treat non-pregnant smokers [31], our analysis suggests that the ability of English SSSP to engage with and support smokers was increased in more deprived areas where a higher proportion of pregnant smokers lived.

It is worth considering how NHS SSSP provision could be altered in response to our findings that SSSP are relatively successful at engaging with deprived pregnant smokers but that deprivation is associated with poorer cessation outcomes. Ideally, SSSP would ‘reach’ large numbers of pregnant smokers and once such smokers were engaged with support, cessation outcomes for deprived smokers would be at least similar and ideally better than those for less deprived women. An audit of Scottish SSSP presented observational data relating SSSP throughput and outcomes with key characteristics; this suggested that ‘opt out’ referral policies, in which all smokers identified during routine antenatal care are referred for SSSP support, [32] might increase service Reach. Subsequently, a before-after study run in one NHS acute trust showed that introducing this kind of referral pathway not only doubled the proportion of women supported by SSSP (i.e. Reach) but it also doubled their chance of short-term quitting (Effectiveness). [33] A simple message for NHS cessation support providers is that ‘opt out’ referral pathways improve both key service outcomes; further research is also needed to determine how cessation outcomes for deprived smokers who receive support can be further improved.

### Conclusions

To our knowledge this is the first study to investigate factors that are influential in national provision of smoking cessation support in pregnancy. For rational use of resources, those introducing similar support programmes in other high-income countries should consider prioritising delivery of support from central locations and using home support visits only in relatively exceptional circumstances. Further work is required to understand how characteristics of individual pregnant women might affect service outcomes, including the likelihood of these women achieving abstinence from smoking. Furthermore, in order to formally test the finding that clinic-based support is more effective, SSSP practices may wish to stratify routinely collected statistics by whether a home or clinic service was provided.

## Abbreviations

CO: Carbon monoxide
DH: Department of Health
DIC: Deviance information criterion
IFS: Infant feeding survey
IMD: Index of multiple deprivation
MCMC: Markov Chain Monte Carlo
MICE: Multiple imputation using chained equations
PCT: Primary care trust
SHA: Strategic health authority
SSS/P: Stop smoking service/for pregnant women
VPC: Variance partition coefficient
WHO: World health organization
